# Classical and Delayed Orthostatic Hypotension in Patients With Unexplained Syncope and Severe Orthostatic Intolerance

**DOI:** 10.3389/fcvm.2020.00021

**Published:** 2020-02-21

**Authors:** Parisa Torabi, Fabrizio Ricci, Viktor Hamrefors, Richard Sutton, Artur Fedorowski

**Affiliations:** ^1^Department of Clinical Sciences, Faculty of Medicine, Clinical Research Center, Lund University, Malmö, Sweden; ^2^Department of Clinical Physiology, Skåne University Hospital, Malmö, Sweden; ^3^Department of Neuroscience, Imaging and Clinical Sciences, Institute for Advanced Biomedical Technologies, “G. D'Annunzio” University, Chieti, Italy; ^4^Department of Internal Medicine, Skåne University Hospital, Malmö, Sweden; ^5^National Heart and Lung Institute, Imperial College, London, United Kingdom; ^6^Department of Cardiology, Skåne University Hospital, Malmö, Sweden

**Keywords:** orthostatic hypotension, syncope, catecholamines, arginine vasopressin, tilt-table test

## Abstract

**Background:** Orthostatic hypotension (OH) is a major sign of cardiovascular autonomic failure leading to orthostatic intolerance and syncope. Orthostatic hypotension is traditionally divided into classical OH (cOH) and delayed OH (dOH), but the differences between the two variants are not well-studied. We performed a systematic clinical and neuroendocrine characterization of OH patients in a tertiary syncope unit.

**Methods:** Among 2,167 consecutive patients (1,316 women, 60.7%; age, 52.6 ± 21.0 years) evaluated for unexplained syncope and severe orthostatic intolerance with standardized cardiovascular autonomic tests including head-up tilt (HUT), we identified those with a definitive diagnosis of cOH and dOH. We analyzed patients' history, clinical characteristics, hemodynamic variables, and plasma levels of epinephrine, norepinephrine, C-terminal-pro-arginine-vasopressin (CT-proAVP), C-terminal-endothelin-1, mid-regional-fragment of pro-atrial-natriuretic-peptide and pro-adrenomedullin in the supine position and at 3-min HUT.

**Results:** We identified 248 cOH and 336 dOH patients (27% of the entire cohort); 111 cOH and 152 dOH had blood samples collected in the supine position and at 3-min HUT. Compared with dOH, cOH patients were older (68 vs. 60 years, *p* < 0.001), more often male (56.9 vs. 39.6%, *p* < 0.001), had higher systolic blood pressure (141 vs. 137 mmHg, *p* = 0.05), had lower estimated glomerular filtration rate (73 vs. 80 ml/min/1.73 m^2^, *p* = 0.003), more often pathologic Valsalva maneuver (86 vs. 49 patients, *p* < 0.001), pacemaker-treated arrhythmia (5 vs. 2%, *p* = 0.04), Parkinson's disease (5 vs. 1%, *p* = 0.008) and reported less palpitations before syncope (16 vs. 29%, *p* = 0.001). Supine and standing levels of CT-proAVP were higher in cOH (*p* = 0.022 and *p* < 0.001, respectively), whereas standing norepinephrine was higher in dOH (*p* = 0.001). After 3-min HUT, increases in epinephrine (*p* < 0.001) and CT-proAVP (*p* = 0.001) were greater in cOH, whereas norepinephrine increased more in dOH (*p* = 0.045).

**Conclusions:** One-quarter of patients with unexplained syncope and severe orthostatic intolerance present orthostatic hypotension. Classical OH patients are older, more often have supine hypertension, pathologic Valsalva maneuver, Parkinson's disease, pacemaker-treated arrhythmia, and lower glomerular filtration rate. Classical OH is associated with increased vasopressin and epinephrine during HUT, but blunted increase in norepinephrine.

## Introduction

Orthostatic hypotension (OH) is the most common manifestation of cardiovascular autonomic dysfunction leading to orthostatic intolerance and syncope. Orthostatic hypotension has multiple etiologies and is traditionally divided into neurogenic or non-neurogenic types. Neurogenic OH is caused by primary neurodegenerative disorders or is secondary to endocrine and autoimmune diseases or renal failure. Non-neurogenic causes include drugs, volume depletion, venous pooling, and heart failure (1).

The prevalence of OH increases with advancing age and comorbidities, such as diabetes, Parkinson's disease or kidney failure, ranging from around 3% in younger individuals, to 35% and more in individuals above 75 years (1). In the majority of patients, OH is asymptomatic, but it can cause symptoms of cerebral hypoperfusion, such as dizziness, fatigue, head and neck pain, nausea, visual disturbance, and ultimately syncope. Symptoms of OH are present when there is a critical reduction of mean arterial pressure and cerebral perfusion (1, 2).

Studies have shown that OH is independently associated with increased mortality, cardiovascular events, incident heart failure, atrial fibrillation, and renal failure (2, 3).

Orthostatic hypotension is clinically classified into classical OH (cOH) and delayed OH (dOH). Classical OH is defined as a sustained decrease in systolic blood pressure (SBP) ≥20 mmHg and/or diastolic blood pressure (DBP) ≥10 mmHg, within 30 s−3 min of active standing or head-up tilt (HUT). In dOH, the progressive fall in systolic blood pressure occurs after 3 min (1). Delayed OH was first described by Streeten and Anderson (4) but there is limited data on the pathophysiology of dOH and the differences between classical and delayed OH.

Given the scarcity of data, we aimed to perform a systematic clinical, hemodynamic, and neuroendocrine characterization of patients presenting with OH in a tertiary syncope unit.

We asked the following questions: what are the differences in clinical characteristics between cOH and dOH? Do patients with cOH and dOH have detectable neuroendocrine and hemodynamic differences during supine rest and HUT?

## Materials and Methods

### Patient Population

The present study is a part of the previously described SYSTEMA project (5, 6) and was conducted from September 2008 through October 2018. Briefly, patients with unexplained syncope or severe orthostatic intolerance were referred to the tertiary syncope unit at Skåne University Hospital in Malmö from hospitals and outpatient care in southern Sweden.

**Figure 1:**
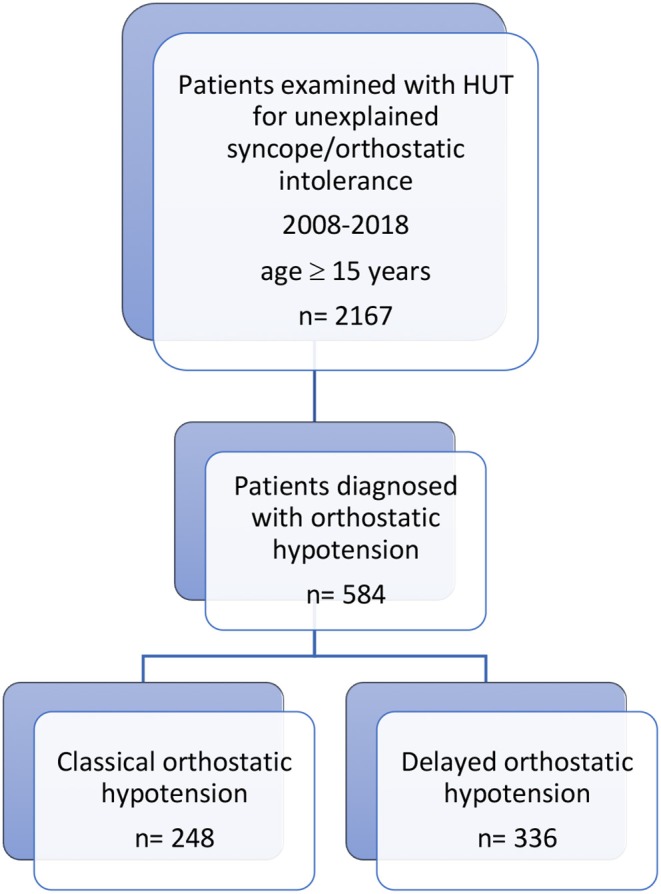
Study design and patient selection process. From the cohort of patients investigated in 2008–2018 for unexplained syncope/orthostatic intolerance with head-up tilt testing (HUT), 2,167 consecutive patients were included. Of these, 584 were diagnosed with orthostatic hypotension, 248 with classical orthostatic hypotension (cOH) and 336 with delayed orthostatic hypotension (dOH). Of these, 111 with cOH and 152 with dOH had blood samples collected both in supine position and after 3 min of HUT and were included in the analysis of neuroendocrine hormones.

The definition of unexplained syncope was a transient loss of consciousness without an established diagnosis after the initial evaluation according to the current syncope guidelines (7, 8). During the study period, 2,167 consecutive patients (1,316 women, 60.7%; age, 52.6 ± 21.0 years) were enrolled. We identified 248 cOH and 336 dOH patients (27% of the entire cohort); of these 111 with cOH and 152 with dOH had blood samples collected both in supine position and at 3 min of HUT as per the initial SYSTEMA study protocol (2008–2014) (6). The flowchart of the study is illustrated in Figure 1.

### Examination Protocol

Patients were requested to take their regular medications and fast for 2 h before the test but were allowed to drink water. A self-administered questionnaire was used to collect data about past medical history and the characteristics of syncope-related symptoms.

The examination included basic cardiovascular autonomic testing (Valsalva maneuver and active standing) and a standard HUT according to the Italian protocol (9) i.e., a drug-free HUT phase of 20 min or until syncope occurred. If the drug-free phase was negative, 400 μg sublingual nitroglycerin was administered and the patient was monitored for another 15 min. This part of the protocol was reserved for patients with unexplained syncope, in whom the passive phase was inconclusive and standing systolic BP was over 90 mmHg. The hemodynamic response during the drug-potentiated HUT phase was not a part of OH evaluation. Classical OH was defined as a sustained decrease in SBP ≥20 mmHg or DBP ≥10 mmHg during first 3 min of head-up tilt (HUT), whereas dOH as SBP/DBP fall ≥20/10 mmHg occurring first after 3 min of tilt testing (10), excluding the obvious pattern of vasovagal reflex i.e., typical prodrome and bradycardia preceding or coinciding with a significant BP fall. A pathologic Valsalva maneuver was defined as the absence of an increase in heart rate during phase II, absence of late phase II blood pressure recovery and delayed blood pressure recovery without the characteristic overshoot during phase IV (7).

All disputable or difficult to interpret cases were resolved by adjudication involving one syncope and autonomic expert, and the examining physician.

Beat-to-beat blood pressure and electrocardiogram were monitored continuously by a validated non-invasive method (Nexfin monitor; BMEYE, Amsterdam, Netherlands or Finapres Nova, Finapres Medical Systems, PH Enschede, Netherlands) (11, 12).

Blood samples were collected from an intravenous line, both in supine position and at 3 min of HUT. The decision to collect blood at 3 min of HUT was based on previous studies (13) indicating that the central blood volume displacement to the lower body, heart rate, and total peripheral resistance reach a steady state at 3 min of orthostasis, which implies that the initial neuroendocrine responses are then fully developed.

### Neuroendocrine Measurements

Measurement of plasma neuroendocrine concentrations has previously been described (6). In brief, we analyzed blood samples in supine position and at 3 min of HUT concentrations of epinephrine, norepinephrine, and non-active peptides C-terminal-pro-arginine-vasopressin (CT-proAVP), C-terminal- endothelin-1 (CT-proET-1), mid-regional-fragment of pro-atrial-natriuretic-peptide (MR-proANP), and mid-regional-fragment of pro-adrenomedullin (MR-proADM). The non-active peptides are generated from the pro-neuroendocrine molecule in ratio 1:1 to the active neuropeptide and are more stable and suitable for analysis compared with their corresponding biologically active substances. High-performance liquid chromatography with fluorescence detection method was used for epinephrine and norepinephrine (14). For measurement of CT-proAVP, CT-proET-1, MR-proANP, and MR-proADM, ThermoScientific BRAHMS assays (BRAHMS GmbH, ThermoFisher Scientific, Neuendorfstrasse 25, 16761 Hennigsdorf, Germany) were used according to the manufacturer's instructions (15, 16).

The regional ethical review board in Lund, Sweden approved the study protocol (reference no 82/2008). All study participants gave written informed consent.

### Statistical Analysis

The main characteristics of the study population are presented as mean and standard deviation for normally distributed continuous variables, as median and interquartile range for non-normally distributed variables and percentages for categorical variables. Intergroup differences were analyzed using ANOVA test for non-categorical variables and Pearson's chi-square test for categorical variables. Neuroendocrine concentrations were log-transformed and standardized (expressed per 1 standard deviation). Logistic regression model adjusted for age and gender was applied to test inter-group differences in neuroendocrine concentrations.

Statistical analyses were carried out using IBM SPSS Statistics version 25 (SPSS Inc., Chicago, IL, USA). All tests were two-sided and *p* < 0.05 was considered significant.

## Results

The main characteristics of the study population are presented in Table 1.

**Table 1:** Clinical characteristics of the study population.

**Characteristic**	**All** ***n* = 584**	**Classical** **OH** ***n* = 248**	**Delayed** **OH** ***n* = 336**	***P*-value**
Age, years	64 ± 18	68 ± 14	60 ± 20	**<0.001**
Sex (male), *n* (%)	274 (47)	141 (57)	133 (40)	**<0.001**
Height (cm)	172 ± 10	173 ± 10	171 ± 10	**0.004**
Body mass index (kg/m^2^)	25 ± 4	25 ± 4	26 ± 5	0.121
Palpitations before syncope, *n* (%)	110 (23)	31 (16)	79 (29)	**0.001**
History of orthostatic dizziness, *n* (%)	439 (76)	183 (74)	256 (77)	0.439
History of syncope, *n* (%)	527 (90)	220 (89)	307 (91)	0.270
Nr of previous syncope episodes (median, interquartile range)	4 (2–10)	4 (2–8)	4 (2–10)	0.160
History of falls, *n* (%)	319 (55)	137 (56)	182 (55)	0.502
Atrial fibrillation, *n* (%)	76 (13)	29 (12)	47 (14)	0.437
History of coronary artery disease, *n* (%)	62 (11)	28 (11)	34 (10)	0.649
Pacemaker therapy, *n* (%)	20 (3)	13 (5)	7 (2)	**0.04**
Parkinsons disease, *n* (%)	16 (3)	12 (5)	4 (1)	**0.008**
Supine systolic blood pressure, mmHg	139 ± 24	141 ± 26	137 ± 22	0.051
Supine diastolic blood pressure, mmHg	74 ± 12	75 ± 12	73 ± 11	0.155
Supine heart rate, beats/min	70 ± 12	69 ± 12	70 ± 12	0.272
Lowest systolic blood pressure during HUT, mmHg	95 ± 22	88 ± 22	99 ± 20	**<0.001**
Lowest diastolic blood pressure during HUT, mmHg	60 ± 13	56 ± 13	63 ± 12	**<0.001**
Max heart rate during HUT (beats/min)	83 ± 16	82 ± 17	85 ± 16	0.069
Pathologic Valsalva maneuver, *n* (%)	135 (29)	86 (43)	49 (18)	**<0.001**
Estimated GFR (mL/min/1.73 m^2^)	77 ± 22	73 ± 21	80 ± 22	**0.003**
Reduced ejection fraction, *n* (%)	119 (21)	42 (18)	77 (24)	0.068
Diabetes, *n* (%)	59 (10)	25 (10)	34 (10)	0.969
Use of betablockers, *n* (%)	153 (26)	62 (25)	91 (27)	0.519
Use of calcium channel blockers, n (%)	96 (17)	38 (15)	58 (18)	0.508
Use of RAAS-antagonists *n* (%)	95 (16)	44 (18)	51 (15)	0.450
Use of loop-diuretics, *n* (%)	63 (11)	32 (13)	31 (9)	0.171
Use of alphablockers, *n* (%)	27 (5)	16 (6)	11 (3)	0.075

*OH, orthostatic hypotension; GFR, glomerular filtration rate; RAAS, renin-angiotensin-aldosterone system. Data are presented as mean ± SD unless indicated otherwise*.

*Values in bold are significant (p > 005)*.

**Figure 2:**
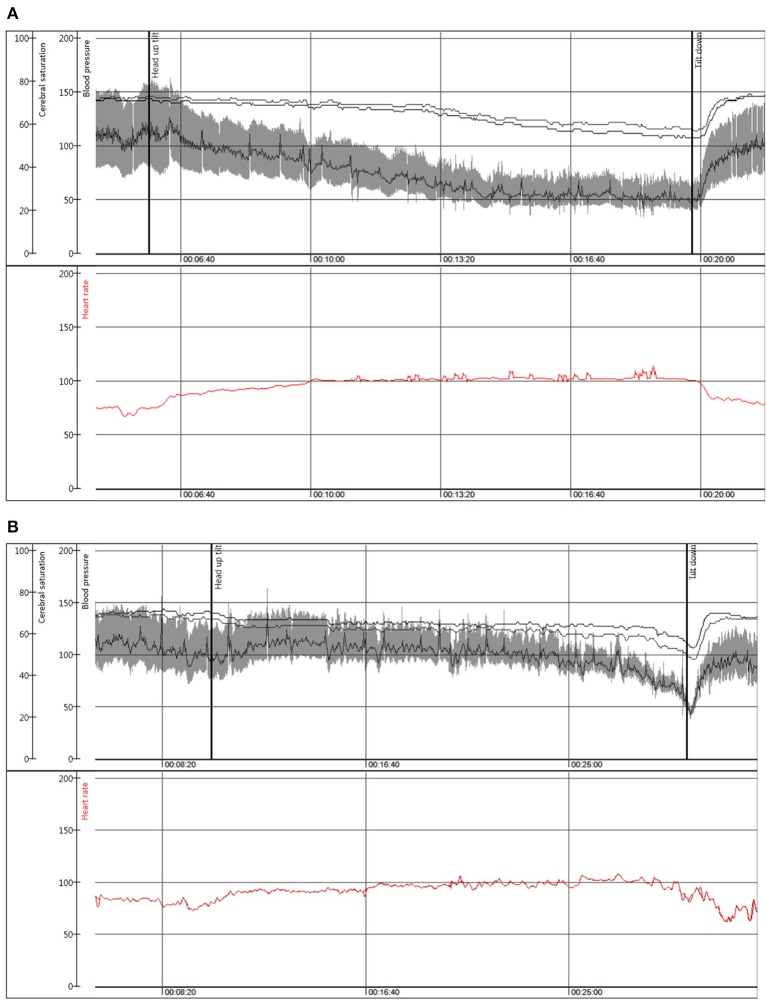
Beat to beat blood pressure (mmHg), and cerebral oxygen saturation (%) in the upper panel with heart rate (beats/min) depicted in red in the lower panel during head-up tilt in a representative patient with **(A)** classical orthostatic hypotension, woman, 50 years; **(B)** delayed orthostatic hypotension leading to an onset of vasovagal reflex and syncope, man, 80 years.

Compared with dOH, patients with cOH were older (68 vs. 60 years, *p* < 0.001), taller (173 vs. 171 cm, *p* = 0.004), more likely men (56.9 vs. 39.6%, *p* < 0.001), had higher supine blood pressure (141 vs. 137 mmHg, *p* = 0.05), lower estimated glomerular filtration rate (73 vs. 80 ml/min/1.73 m^2^, *p* = 0.003), reported less palpitations before syncope (16 vs. 29%, *p* = 0.001), more often had a pathologic Valsalva maneuver (86 vs. 49 patients, *p* < 0.001), a pacemaker (5 vs. 2%, *p* = 0.04), and Parkinson's disease (5 vs. 1%, *p* = 0.008). Patients with cOH had a more pronounced fall in systolic (88 vs. 99 mmHg, *p* < 0.001) and diastolic (56 vs. 63 mmHg, *p* < 0.001) blood pressure during HUT than those with dOH. Figure 2 displays typical hemodynamic responses during HUT in the two forms of OH.

Clinical characteristics that did not differ between the cOH and dOH groups were history of falls, history of previous syncope and number of previous syncope episodes, diagnosis of atrial fibrillation, supine heart rate, maximum heart rate during HUT, occurrence of reduced ejection fraction (below 55%), coronary artery disease, and medications (betablockers, calcium channel blockers, RAAS-antagonists, loop-diuretics, alpha-blockers).

The plasma levels of neuroendocrine markers are shown in Table 2. Supine and 3-min HUT levels of CT-proAVP were higher in cOH compared with dOH (*p* = 0.022 and *p* < 0.001). In contrast, at 3 min of HUT, norepinephrine was higher in dOH (*p* = 0.001). Further, Δepinephrine (*p* < 0.001) and ΔCT-proAVP (*p* = 0.001) (i.e., 3 min of HUT minus supine values) were higher in cOH, whereas Δnorepinephrine was higher in dOH (*p* = 0.045) compared with cOH.

**Table 2:** Plasma concentrations of assessed neuroendocrine hormones.

**Hormone**	**All** ***n* = 263**	**Classical OH** ***n* = 111**	**Delayed OH** ***n* = 152**	*P*-value^*^
Epinephrine (0) (nmol/L)	0.15 (0.09–0.24)	0.14 (0.09–0.21)	0.15 (0.09–0.24)	0.954
Epinephrine (3) (nmol/L)	0.22 (0.08–0.35)	0.22 (0.13–0.44)	0.22 (0.13–0.32)	0.128
Norepinephrine (0) (nmol/L)	2.30 (1.60–3.10)	2.20 (1.50–3.10)	2.40 (1.70–3.20)	0.263
Norepinephrine (3) (nmol/L)	3.40 (1.80–4.60)	3.10 (1.90–4.70)	3.60 (2.63–4.56)	**0.001**
CT-proAVP (0) (pmol/L)	8.18 (4.53–14.4)	9.42 (6.01–16.3)	7.30 (3.94–12.3)	**0.022**
CT-proAVP (3) (pmol/L)	8.38 (4.57–16.1)	10.5 (6.24–22.5)	6.55 (3.77–12.4)	**<0.001**
CT-proET-1 (0) (pmol/L)	61.8 (50.8–74.2)	63.0 (52.2–74.6)	61.1 (49.9–70.8)	0.406
CT-proET-1 (3) (pmol/L)	56.0 (43.2–71.1)	58.8 (44.5–72.5)	55.0 (41.9–67.4)	0.908
MR-proANP (0) (pmol/L)	109 (65.9–161)	109 (67.3–150)	109 (61.3–168)	0.940
MR-proANP (3) (pmol/L)	109 (61.6–152)	111 (64.6–150)	108 (58.3–173)	0.758
MR-proADM (0) (pmol/L)	0.68 (0.50–0.91)	0.74 (0.50–1.06)	0.63 (0.50–0.85)	0.132
MR-proADM (3) (pmol/L)	0.59 (0.42–0.83)	0.64 (0.44–1.00)	0.56 (0.40–0.81)	0.233

*The concentrations of neuroendocrine hormones given as median (interquartile range) for supine (0) and 3 min HUT (3). CT-proAVP indicates C-terminal-pro-arginine-vasopressin, CT-proET, C-terminal-endothelin-1, MR-proANP, mid-regional-fragment of pro-atrial-natriuretic-peptide and MR-proADM mid-regional-fragment of pro-adrenomedullin*.

* *P-values for log-transformed concentrations*.

*Values in bold are significant (p > 005)*.

The plasma levels of CT-proET-1, MR-proANP, and MR-proADM were not different between the cOH and dOH groups.

## Discussion

This study examined the differences in clinical characteristics, hemodynamic variables, and plasma concentrations of neuroendocrine hormones in patients with classical and delayed orthostatic hypotension. We found that patients with classical OH were older, more likely men, more often hypertensive, treated with pacemaker, had lower kidney function and more often pathologic Valsalva test, Parkinson's disease, and reported less palpitations before syncope. Compared with delayed OH, classical OH was associated with increased levels of vasopressin and epinephrine during HUT, whereas norepinephrine increased more during HUT in delayed OH.

Moving from supine to standing position causes an immediate redistribution of up to 1 l of blood to the capacitance vessels of the legs and splanchnic/pelvic circulation. This prompts a decrease in venous return and cardiac output, which, in the healthy individual, is counteracted by the baroreflex, causing increased sympathetic outflow and vagal inhibition. Heart rate, cardiac contractility, and peripheral vascular resistance increase to maintain blood pressure. During prolonged standing, transcapillary fluid filtration into the interstitial space can reduce plasma volume up to 20% (17). Neuroendocrine responses, primarily mediated by the renin-angiotensin-aldosterone system cause volume expansion and become important during prolonged orthostatic stress. Vasopressin-secretion by the hypothalamus has a smaller role in maintaining blood pressure in the normovolemic state in healthy individuals (18).

In patients with autonomic dysfunction, norepinephrine release from postganglionic sympathetic nerves is reduced or insufficient, causing inadequate peripheral arteriolar vasoconstriction and subsequent hypotension during orthostasis (17). This process may be associated with compensatory mechanisms governed by different neuroendocrine systems.

### Neuroendocrine Changes and Orthostatic Hypotension

#### Vasopressin

Vasopressin is released in response to hyperosmolarity and reduced arterial pressure, leading to renal water reabsorption and vasoconstriction (19). Vasopressin acts as a backup system to the renin-angiotensin and sympathetic systems, and is not critical for hemodynamic stability as long as the former are intact (19). Blockade of V1 receptors caused a larger fall in blood pressure in patients with diabetic autonomic dysfunction compared with controls, showing that these patients are more dependent on vasopressin to maintain blood pressure (20).

Vasopressin levels increased 3-fold in healthy subjects during HUT (21, 22) and a similar increase was seen in patients with autonomic failure and falling blood pressure on HUT, but the increase was not sufficient compared with the magnitude of blood pressure fall (21). Studies have shown that the subnormal vasopressin response in patients with autonomic dysfunction is caused by a defect in the baroreflex arc (23, 24).

Our findings are consistent with previous studies. Patients with cOH had more pronounced falls in systolic and diastolic blood pressure than those with dOH. The supine and standing levels of CT-proAVP were significantly higher in cOH compared to dOH. The fall in blood pressure and right atrium filling stimulates vasopressin secretion. Vasopressin was measured after 3 min of HUT, when the hemodynamic impairment is fully developed in cOH but not in dOH. Consequently, vasopressin response after 3 min of standing was greater in cOH.

#### Epinephrine

In healthy volunteers, epinephrine initially increased 3-fold during standing, and normalized after prolonged orthostasis (22). In a study comparing older OH males with controls, no difference was seen in epinephrine concentrations at the end of HUT (25).

We found that the change in epinephrine concentration at 3 min of HUT was more pronounced in cOH compared with dOH. A variety of stressors induce higher plasma epinephrine increases than those in norepinephrine, indicating a greater adrenomedullary than noradrenergic system response (26). Since blood pressure fall during HUT was higher for cOH than dOH, it is reasonable to assume that a steeper orthostatic blood pressure fall would evoke a more significant epinephrine response at 3 min of HUT. Moreover, the compensatory release of epinephrine through adrenomedullary mechanisms might be more pronounced in the presence of impaired function of the autonomic nervous system due to neurodegenerative processes affecting the release of norepinephrine from neural endings (27).

#### Norepinephrine

In healthy subjects, plasma norepinephrine concentration doubles within 5 min of standing (22). Orthostatic hypotension is associated with impaired norepinephrine release (27, 28) whereas in dOH norepinephrine levels are normal or increased (4).

Consistent with previous studies, we found that orthostatic norepinephrine concentration was higher in dOH and that it increased more on HUT compared with cOH. These data would indicate dOH as a milder form of sympathetic autonomic dysfunction compared with cOH.

### Clinical Features of Orthostatic Hypotension

Consistent with previous reports, we found that patients with dOH were younger than those with cOH, supporting the idea that dOH might be an early presentation of autonomic dysfunction, preceding cOH (29). Another finding that supports this is that cOH patients more often had a pathologic Valsalva maneuver than dOH patients. It has been reported that cOH is associated with more severe autonomic dysfunction compared with dOH (30). Conditions that that are associated with autonomic dysfunction and OH, such as Parkinson's disease (31, 32) and reduced renal function (33, 34), were also more common in the cOH group. Surprisingly, diabetes, which is a common cause of secondary OH, was equally prevalent in the two groups.

Orthostatic hypotension is frequently associated with supine hypertension (35, 36). Goldstein found that OH was associated with supine hypertension in patients with primary neurodegenerative diseases. These patients also had low plasma concentrations of norepinephrine, indicating that the supine hypertension in OH is caused by other mechanisms than the hyperactivation of the sympathetic nervous system (35). The observation that cOH patients more frequently have supine hypertension implies that cOH is associated with more severe abnormalities of both autonomic and neuroendocrine control mechanisms.

Studies have shown that cOH is associated with increased risk of cardiovascular disease (3, 37), which can explain the higher incidence of pacemaker treatment in cOH patients. Autonomic dysfunction with sympathetic denervation of the heart that caused sick sinus syndrome has been reported (38). Cardiac denervation could be a possible explanation for the observation that cOH patients reported less palpitations before syncope.

Delayed OH was first described by Streeten and Anderson (4) and has been viewed as a benign condition, but more recent studies indicate otherwise. Gibbons and Freeman followed individuals diagnosed with cOH and dOH for 10 years and discovered that 54% of patients with dOH eventually developed cOH, and 31% developed alpha-synucleopathies (39). Mortality in dOH was increased compared with controls, but not as high as in cOH. Such long-term data indicate dOH not to be a benign non-progressive condition, but in the majority of cases, is an early presentation of cOH.

Better characterization of classical and delayed orthostatic hypotension can have implications for diagnosis, prevention, and treatment of these conditions. In clinical practice, delayed orthostatic hypotension is often overlooked although there are similar clinical consequences in both variants such as syncope as indicated by this study. Whether delayed and classical orthostatic hypotension represent a sequential worsening of the same disease, or constitute two different entities of cardiovascular autonomic dysfunction with different therapeutic approaches, warrants further research.

## Study Limitations

There are several important limitations that should be acknowledged. One is that this a single-center study and another is that there is no healthy control group. Vasoactive medications were not withdrawn before HUT as the tests were performed to detect syncope etiology in the real-life scenario, meaning without discontinuing the regular medication. The amount of water ingested prior to HUT, which may have potentially introduced a bias in the measured neurohormone levels, was not recorded. Neuroendocrine hormones were only measured in supine position and at 3 min of HUT. Other changes in the levels of these hormones might have occurred after prolonged orthostasis. Other neuroendocrine hormones that were not included in this study may have a significant role in OH pathophysiology. Finally, plasma norepinephrine concentration represents the spillover from synapses. The concentration is dependent on both the release into plasma and removal by reuptake into nerve terminals and might not accurately reflect the rate of sympathetic nerve traffic.

## Conclusion

Compared with delayed orthostatic hypotension, patients with the classical form are older, more often have supine hypertension, autonomic dysfunction, Parkinson's disease, pacemaker-treated arrhythmia, and lower glomerular filtration rate. Classical orthostatic hypotension is associated with increased levels of vasopressin and epinephrine during head-up tilt, but blunted increase in circulating norepinephrine. These findings suggest that classical orthostatic hypotension, compared with the delayed form, is associated with more severe abnormalities of both autonomic and neuroendocrine control mechanisms and can be regarded as a more advanced and severe condition.

## Data Availability Statement

The datasets generated for this study are available on request to the corresponding author.

## Ethics Statement

The studies involving human participants were reviewed and approved by Regional Ethical Review Board in Lund (82/2008). The patients/participants provided their written informed consent to participate in this study.

## Author Contributions

All the coauthors conceived and designed the study. PT, AF, FR, and VH reviewed the literature. RS gave a critical input for the analyses. PT, AF, and VH collected the data for the study. PT, AF, and FR performed the statistical analyses. PT and AF drafted the manuscript. FR, VH, and RS performed the critical review of the manuscript. All coauthors accepted the final version. AF provided the founding for the study.

### Conflict of Interest

AF reports personal fees from Medtronic Inc. and Biotronik outside the submitted work; RS reports personal fees and other from Medtronic Inc., St. Jude Medical Inc. (Abbott Laboratories) outside the submitted work; RS is a member of the speaker's Bureau St. Jude Medical/Abbott Inc.; RS is shareholder in Boston Scientific Inc., Edwards Lifesciences Inc. and AstraZeneca PLC. The remaining authors declare that the research was conducted in the absence of any commercial or financial relationships that could be construed as a potential conflict of interest.
